# Determinants of the intention to work in aged care: a cross-sectional study to assess gerontological nursing competencies among undergraduate nursing students

**DOI:** 10.1186/s12912-023-01613-1

**Published:** 2023-11-29

**Authors:** Mu-Hsing Ho, Jung Jae Lee, Jee Young Joo, Kasia Bail, Megan F. Liu, Victoria Traynor

**Affiliations:** 1 School of Nursing, LKS Faculty of Medicine, The University of Hong Kong, Pokfulam, Hong Kong SAR; 2 College of Nursing, Gachon University, Incheon, Korea; 3 Faculty of Health, Australian Capital Territory, University of Canberra, Canberra, Australia; 4 School of Gerontology and Long-Term Care, College of Nursing, Taipei Medical University, Taipei, Taiwan; 5 School of Nursing, Faculty of Science, Health and Medicine, University of Wollongong, Wollongong, NSW Australia

**Keywords:** Aged, Competency-based education, Nursing, Long-term care, Intention

## Abstract

**Background:**

There are huge demands for aged-care workers, and undergraduate gerontological nursing education plays a critical role in providing academic and professional training.

**Purpose:**

To examine relationships of characteristics, aged-care education, and gerontological nursing competencies with the intention to work in aged care.

**Methods:**

An online survey was distributed to undergraduate nursing students between April and June 2022 to gather characteristics, relevant aged-care education, gerontological nursing competencies, and intentions to work in aged care data. Multivariate regression analyses were conducted to identify determinants of the intention to work in aged care.

**Results:**

Students (n = 358) who were older (*p* < 0.001) and who were married (*p* < 0.001) had higher intentions to work in aged care. “Promoting mental health and psychological well-being”, “Providing evidence-based dementia care”, and “Enabling access to technology”, were also associated with positive intentions.

**Conclusions:**

This study provides evidence on determinants of the intention to work in aged care, particularly gerontological nursing competencies.

**Supplementary Information:**

The online version contains supplementary material available at 10.1186/s12912-023-01613-1.

## Introduction

Regarding the relevance and importance for a nursing profession in aged care, education in gerontological nursing is one of the most discussed topics in nursing curriculum [1]. Particularly in Asian countries and regions, the rapid ageing population is likely to impact on healthcare service, caregiving burden and healthcare education [2]. Aged-care in nursing education and relevant curriculum is crucial to provide professional skills and competencies for nursing students to be equipped. Nevertheless, acute, medical, and surgical care settings dominate traditional undergraduate nursing curriculum designs, whereas geriatric and gerontological aspects are often ignored [3, 4].

## Literature review

Undergraduate gerontological nursing education plays a critical role in providing academic and professional training. To guarantee that students have the information, abilities, and positive attitudes necessary to care for older persons in order to fulfill the requirements of the world’s rapidly ageing population, undergraduate gerontological nursing education is essential [5, 6]. However, the inadequacy and inconsistencies of aged education have significant impacts on the quality and safety of aged care [7]. Aged-care nurses working in hospitals, senior communities, and nursing homes must be able to adequately provide care for older adults with complicated health conditions and comorbidities. This is a specialty area of practice, and therefore nurses working in aged care must receive proper training and education to understand gerontological nursing competencies to ensure they provide skilled care [8].

There are significant international efforts to define and implement gerontological nursing competencies and nursing standards [9–13]. In Australia, a comprehensive gerontological nursing competencies framework was developed, which includes 33 aspects of practice that cover everyday activities and responsibilities of aged-care nurses [14]. The gerontological nursing competencies framework was designed by a research team that worked with the Department of Health and Aged Care in Australia and secured federal government funding for developing a Gerontological Nurse Competencies program [15]. The gerontological nursing competencies framework also provides guidance in (1) law, ethics, and decision-making; (2) provision of palliative care; and (3) enabling access to technology, e-health, and social media, which were not covered by existing competency frameworks [14]. The use of assistive technology, e-health, and m-health is commonplace for healthcare access worldwide, including aged care [16, 17]. Studies also evaluated the mental health and psychological well-being, quality of death, and end-of-life concerns among older adults in long-term care settings [18, 19].

Evidence showed that students have significant improvements in knowledge, attitudes, and working intentions towards aged care if they attend a stand-alone gerontological and geriatrics course as well as clinical placement in their undergraduate nursing education program [6]. This highlights the importance of aged-care education implemented in nursing curricula, which could highly impact students’ working intentions regarding employment in aged care. However, no such comprehensive gerontological nursing competency framework has been developed and implemented to investigate the level of gerontological nursing competencies of nursing students. As there are huge demands for aged-care workers, understanding nursing students’ intentions to work in aged care and their determinants is of particular importance. Therefore, this study aimed to examine relationships of personal characteristics, aged-care education, and gerontological nursing competencies with intentions to work in aged care among nursing students.

## Methods

### Design

This was a cross-sectional, descriptive study reporting determinants of the intention to work in aged care among nursing students in Taiwan.

### Participants and data collection

Participants were mainly recruited through an online survey between April and June 2022 in Taiwan. The selection criteria were nursing students aged older than 18 years. We targeted students in both the Bachelor of Science in Nursing and Post-Baccalaureate Program in Nursing programs to understand associations of personal characteristics and gerontological nursing competencies with intentions to work in aged care. The survey was distributed using a web-based survey tool (Qualtrics, Provo, UT, USA) to collect information from participants. Invitations with a QR code were sent to teaching staff at universities in Taiwan and were promoted on social media services such as webpages of nursing students on Facebook, Instagram, and Twitter. The title page of the online survey provided information regarding the study aims and data use and storage. Participants were also fully informed that the survey was anonymous and that all collected data would be de-identified. Completion of the survey implied informed consent. We conducted a sample size estimation using G* power version 3.0.10 software [20]. The statistical test and model settings for the sample size estimation were as follow: *F* tests as the test family; and linear regression: fixed model, *R*^*2*^ deviation from zero (given *α*, power, and effect size *f*). Parameter settings (*α* = 0.05, 1–*β* = 0.90, effect size *f* = 0.05, and number of predictors = 3) were established according to a previous study that investigating gerontological nursing competencies and intentions to work in aged care among aged-care nurses. A total number of 222 samples was suggested. An attrition rate of 30% was considered, and thus an estimated sample size of 317 participants was determined and considered sufficient. The Strengthening the Reporting of Observational Studies in Epidemiology (STROBE) reporting guidelines for cross-sectional studies were followed [21].

### Measurements

The online survey consisted of questions pertaining to (1) basic characteristics of respondents, such as age, gender, degree or program enrolled, marital status, and year of study, (2) relevant aged-care education and additional training they had received on aged care, (3) gerontological nursing competencies, and (4) intention to work in aged care.

### Personal characteristics

Basic characteristics of respondents, such as age, gender, degree or program enrolled, marital status, and year of study were collected.

### Relevant aged-care education and additional training they had received on aged care

A couple of structured questions were generated to understand the aged-care education and curriculum provided by a respondent’s university. We listed course names that could be found publicly at all universities that have a nursing school and included an open-ended question if a course name was not listed in the survey. To identify whether students had received no aged-care course as a common core course in the nursing curriculum, students were also asked if these courses were optional courses, and courses were categorized into common core or optional courses. In addition, they were asked if the school/university provided any additional training (e.g., workshops, seminars, short courses during clinical placement) on aged care through open-ended questions. Responses were categorized into dementia, physical well-being (e.g., screening for frailty and sarcopenia in older adults), hospice and palliative care, nutrition, and psychological well-being (e.g., mental health, depression, anxiety, etc.).

### The gerontological nursing competencies (GNCs) scale

**Fig. 1:**
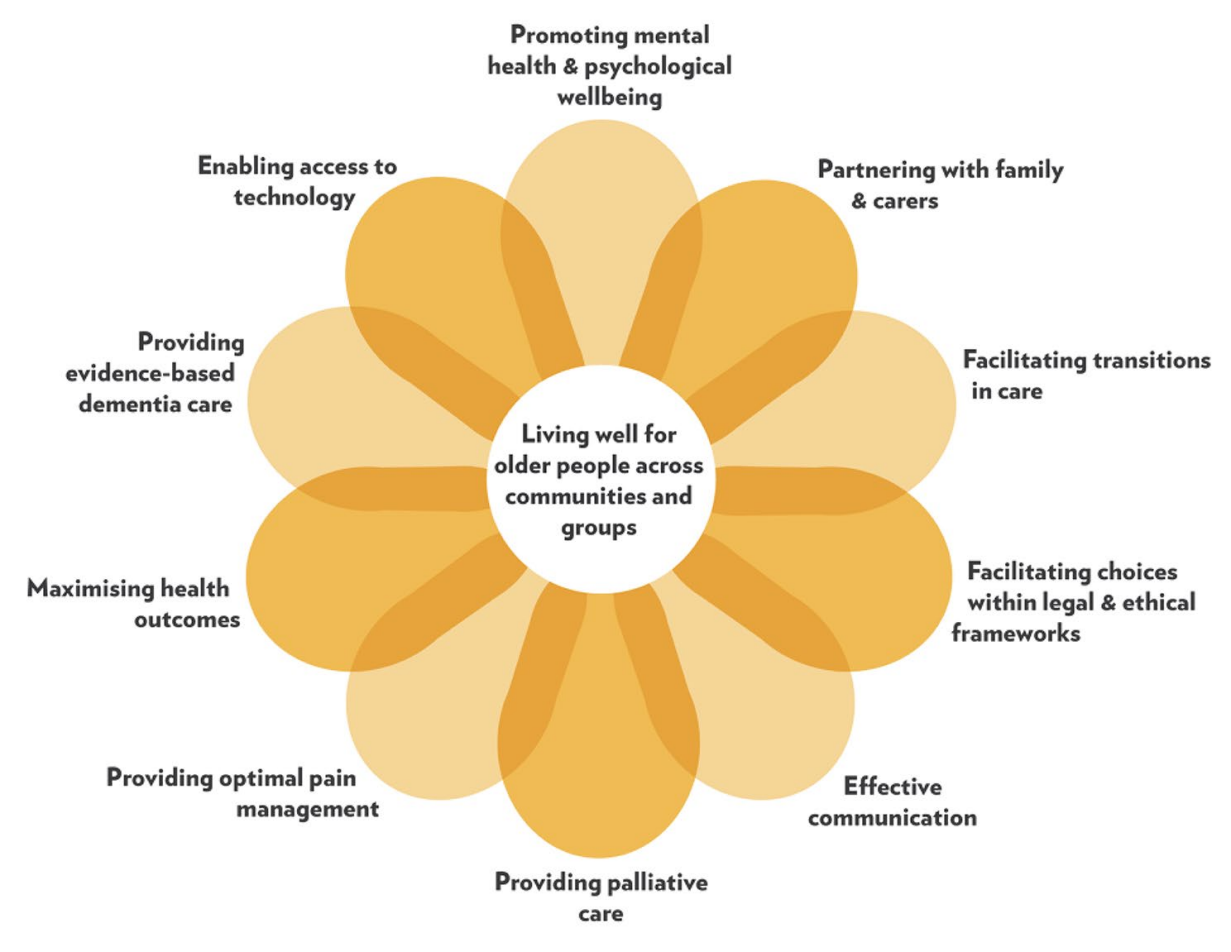
The 11 core competencies of the gerontological nursing competency framework. Adopted from Aged Dementia Health Education & Research (ADHERe) centre (https://adhere.org.au/gerontological-nursing-competencies/)

The gerontological nursing competencies of nursing students were measured by a Mandarin version of the GNCs scale. The GNCs scale consists of 11 core competencies with 33 items to measure the level of perceived gerontological nursing competencies (Fig. 1) [22]. A five-point Likert scale (1 = least competent, 5 = most competent) was used for each item. Two subscales, namely ‘essential’ and ‘enhanced’, measured the perceived competencies in various levels of gerontological nursing practice. The face validity, content validity, and construct validity (which explained 80.774% of the total variance) were established. The internal consistency and test-retest reliability were also confirmed in a psychometric evaluation study done by our team [22]. The English language version of the GNCs scale items can be found in the Supplementary file 1.

### Intention to work in aged care

A simple, single item with a five-point Likert scale rated from 1 (not at all) to 5 (very much) was used to measure a nursing student’s intention to work in aged care among participants.

### Data analysis

SPSS version 25.0 (IBM, Armonk, NY, USA) was used for all analyses. Descriptive statistics including frequencies, percentages, means, and standard deviations (SDs) were used to present the distribution of variables in tables and graphical approaches. The Kolmogorov-Smirnov test was performed to confirm that data were normally distributed. An independent *t*-test, one-way analysis of variance (ANOVA), and Pearson’s correlation tests were used to examine relationships between characteristics and intention to work in aged care. The least significant difference (LSD) technique was used for a post-hoc test in the ANOVA. Multivariate linear regression analyses were conducted to identify GNCs core competencies associated with the intention to work in aged care among nursing students. Characteristics significantly related to the intention to work in aged care were entered into the regression model for adjustment. In the multivariate linear regression, the variance inflation factor (VIF) was confirmed to detect potential multicollinearity (< 10). All tests were two-tailed, and the significance level (*α*) was set to 0.05.

## Results

### Descriptive results

**Fig. 2:**
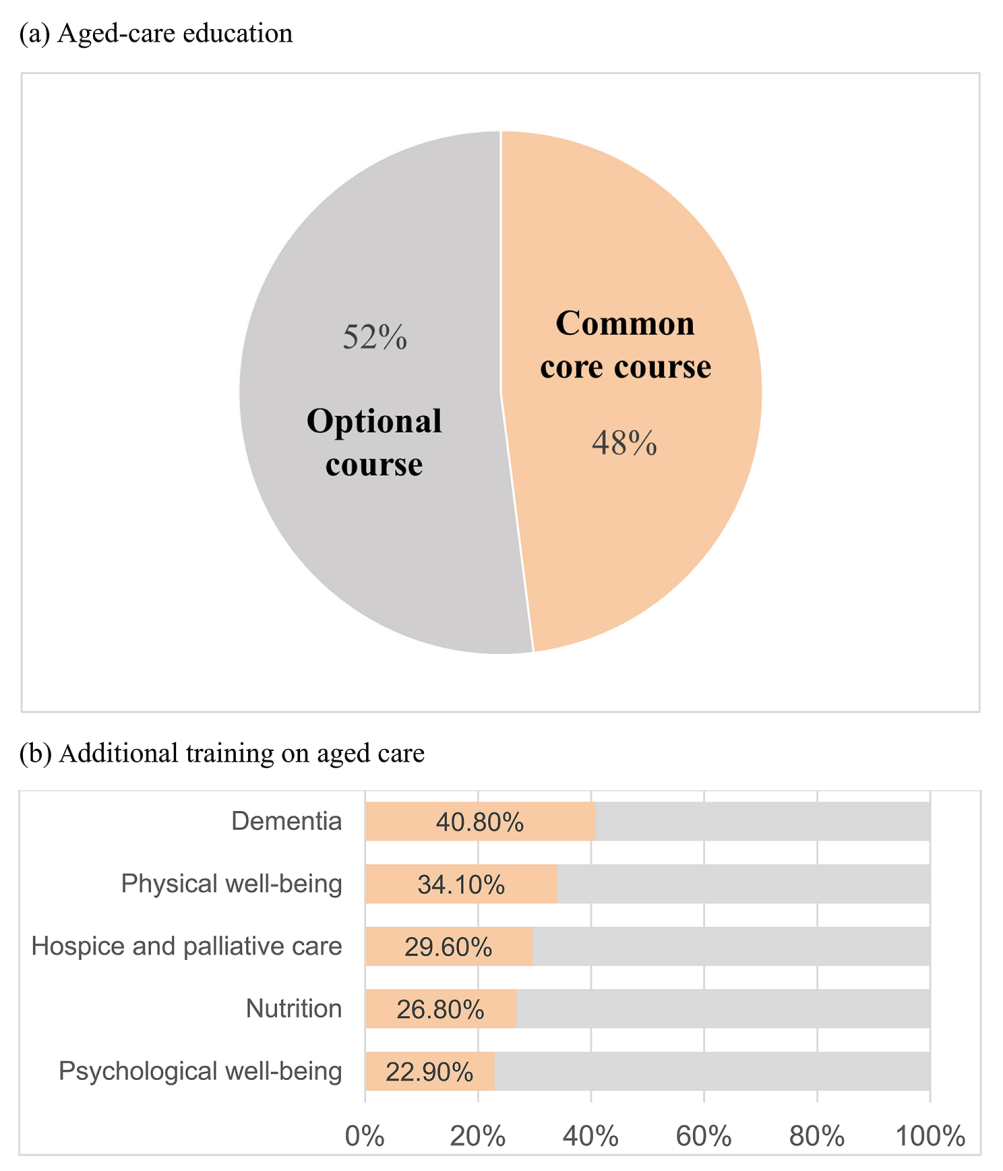
Relevant aged-care education received among nursing students: **(a)** aged-care education and **(b)** additional training on aged care ***Notes.*** Physical well-being (frailty, sarcopenia, etc.) and mental health and psychological well-being (depression, anxiety, etc.)

In total, 358 nursing students completed the online survey and were included in the final analysis. Table 1 shows the basic characteristics of included students. As shown in Fig. 2, only 48% of the nursing curricula included aged-care education as a common core course. Apart from the nursing curriculum, most of the additional training on aged care received by students was dementia care (40.8%), followed by physical well-being (34.1%), hospice and palliative care (29.6%), nutrition (26.8%), and psychological well-being (22.9%). The most competent aspect of gerontological nursing was “Communicating effectively”, and the least competent aspect was “Facilitating choices within legal and ethical frameworks” (Supplementary file 1).

**Table 1:** Characteristics of the students (*N* = 358)

Characteristic		*n* (%)
Age, mean (SD), years		23.13 (5.23)
	18 ~ 22	247 (69.0)
	≥ 23	111 (31.0)
Gender		
	Female	306 (85.5)
	Male	52 (14.5)
Degree/Program		
	Bachelor of Science in Nursing	269 (75.1)
	Post-Baccalaureate Program in Nursing	89 (24.9)
Marital status		
	Single	340 (95.0)
	Married	18 (5.0)
Year of study		
	Year 1	128 (35.8)
	Year 2	66 (18.4)
	Year 3	100 (27.9)
	Year 4	64 (17.9)

SD: standard deviation

### Relationships between student’s characteristics and intentions to work in aged care

Table 2 presents significant relationships between personal characteristics and intentions to work in aged care. Students who were older (*p* < 0.001) and married (*p* < 0.001) had higher intentions of working in aged care than did younger and single students. In addition, year 1 students had higher intentions of working in aged care than did year 3 and 4 students (*p* = 0.033). No significant differences between relevant aged-care education and additional training received on aged care on the intention were observed.

**Table 2:** Relationships between student’s characteristics and their intention to work in aged care (*N* = 358)

Variable		Mean (SD)	*t*/*F/r*	*p* value	Post-hoc ^a^
Age, years			0.182	**< 0.001**	
	18 ~ 22 (*n* = 247)	3.06 (1.01)	-3.465	**< 0.001**	
	≥ 23 (*n* = 111)	3.51 (1.21)			
Gender					
	Female (*n* = 306)	3.20 (1.09)	0.043	0.483	
	Male (*n* = 52)	3.19 (1.17)			
Degree/program enrolled					
	Bachelor of Science in Nursing (*n* = 269)	3.19 (1.08)	-0.150	0.440	
	Post-Baccalaureate Program in Nursing (*n* = 89)	3.21 (1.16)			
Marital status					
	Single (*n* = 340)	3.16 (1.08)	-2.996	**0.001**	
	Married (*n* = 18)	3.94 (1.11)			
Year of study					
	(1) Year 1 (*n* = 128)	3.42 (1.15)	2.945	**0.033**	(1)>(3) (1)>(4)
	(2) Year 2 (*n* = 66)	3.11 (1.17)			
	(3) Year 3 (*n* = 100)	3.10 (0.89)			
	(4) Year 4 (*n* = 64)	3.00 (1.14)			
Aged-care education					
	Common core course (*n* = 172)	3.28 (1.15)	-1.337	0.091	
	Optional course (*n* = 186)	3.12 (1.04)			
Dementia training					
	No (*n* = 212)	3.13 (1.05)	-1.380	0.084	
	Yes (*n* = 146)	3.29 (1.15)			
Hospice and palliative care training					
	No (*n* = 252)	3.19 (1.07)	-0.209	0.417	
	Yes (*n* = 106)	3.22 (1.16)			
Physical well-being training					
	No (*n* = 236)	3.15 (1.07)	-1.099	0.136	
	Yes (*n* = 122)	3.29 (1.15)			
Mental health and psychological well-being training					
	No (*n* = 276)	3.21 (1.06)	0.259	0.398	
	Yes (*n* = 82)	3.17 (1.21)			
Nutrition training					
	No (*n* = 262)	3.18 (1.07)	-0.648	0.259	
	Yes (*n* = 96)	3.26 (1.17)			

***Notes.***^a^ The least significant difference was used for post-hoc test

### Associations between gerontological nursing competencies and intentions to work in aged care

**Fig. 3:**
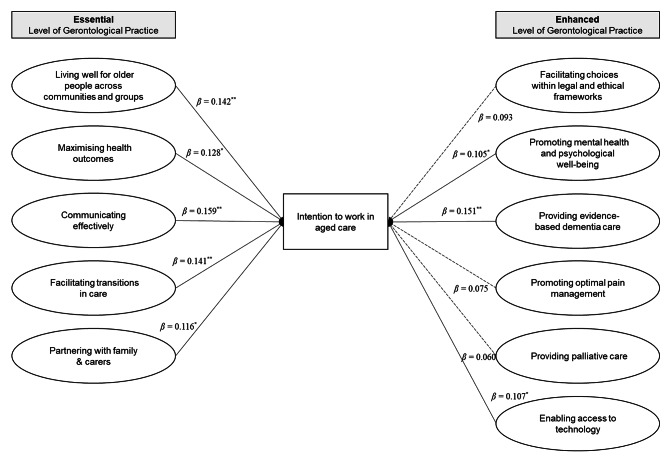
Associations of gerontological nursing competencies with the intention to work in aged care (*N* = 358) ***Notes.*** Adjusted for age, marital status, and year of study. All variance inflation factors were < 2. ^*^p < 0.05, ^**^p < 0.01

After adjusting for age, marital status, and year of study, the multivariate linear regression results showed that all core competencies in the essential level of gerontological practice were significantly associated with an intention to work in aged care. Three core competencies in the enhanced level of gerontological practice, namely “Promoting mental health and psychological well-being”, “Providing evidence-based dementia care”, and “Enabling access to technology”, were also associated with the intention. No multicollinearity was observed in the regression model. Figure 3 provides an overview of associations between gerontological nursing competencies and intentions to work in aged care.

## Discussion

In this study, we report determinants of the intention to work in aged care, particularly the gerontological nursing competencies. Only 48% of nursing curricula were reported to have aged-care education as a common core course. Many schools included “Gerontological/geriatric nursing” courses that cover topics including communication with older adults, assessing nutrition, physical activity, cognitive and mental health as well as spiritual care [23], caregiver burdens, and community care and support (adopted from a course syllabus) [24]. These topics are important and are considered to be core competencies in overall gerontological nursing practice [25].

We found that students with higher GNCs had stronger intentions to work in aged care. Although no significant difference between whether these courses were either common core or optional courses on intentions to work in aged care was observed, increasing the level of gerontological nursing core competencies would likely strengthen students’ intentions to work in aged care. In addition to the nursing curriculum, students also reported that they had received additional training in aged care in terms of dementia care, physical well-being (e.g., frailty and sarcopenia in older adults), hospice and palliative care issues, nutrition in older people, mental health, and psychological well-being. This training was delivered in the form of workshops, seminars, and short courses during clinical placement. Most of this training was considered to be an ‘enhanced’ level of gerontological nursing practice in our gerontological nursing competencies framework. However, we found no significant relationship as to whether or not a participant attended these additional training courses and an intention to work in aged care among nursing students. These results are in line with our further analysis on associations between enhanced levels of gerontological nursing practice and intentions to work in aged care.

The perceived gerontological nursing competencies among students showed that the most competent aspect of gerontological nursing was “Communicating effectively”, and the least competent aspect was “Facilitating choices within legal and ethical frameworks.” The possible reason is that many nursing courses involve essential and advanced communication skills as well as in their clinical placements. Students may have been trained and equipped with the capacity to communicate effectively with patients, particularly older adults. In contrast, facilitating choices within legal and ethical frameworks is not highly emphasized in nursing curriculum [26, 27]. Given our study sample across years 1 to 4, this core competency was still rated as the least competent from students’ perspectives. The importance of ethical and legal education in aged care has been confirmed. Aged-care nurses face many ethical and legal issues, such as ethical conflicts with families, decision-making, and end-of-life issues [28]. This could be further integrated and strengthened in future higher education curriculum development.

In terms of determinants of the intention to work in aged care, students who were an older age and were married had higher intentions to work in aged care. Our study included those who were currently studying in a Post-Baccalaureate Program in Nursing; therefore, some of them already had working experience and an undergraduate degree. This result suggests that strategies for recruiting aged-care nurses could target new graduates of Post-Baccalaureate Programs in Nursing. Having a stronger intention to work in aged care would likely boost the manpower of nursing practice in aged care [29, 30].

As for associations between gerontological nursing competencies and the intention to work in aged care, all core competencies at the essential level of gerontological practice were significantly associated with the intention to work in aged care. This result emphasizes that increasing essential levels of gerontological nursing competencies might also increase the intention to work in aged care. As mentioned above, almost all essential-level gerontological nursing competencies are included in ‘Gerontological/geriatric nursing’ courses, and just over half (52%) were optional curses. We suggest that integrating aged-care courses as common core courses may increase the gerontological nursing competencies and nursing students’ intention to work in aged care. Furthermore, our study utilized a gerontological nursing competencies framework which can provide aspects and topics that could be incorporated in aged-care courses. For example, enhanced levels of gerontological practice, namely “Promoting mental health and psychological well-being”, “Providing evidence-based dementia care”, and “Enabling access to technology”, were positively associated with intentions to work in aged care. Increased uses of technology, such as e-Health, m-Health and social media, have been designed in the development of nursing interventions [16, 17, 31].

Relevant digital literacy and competencies of nursing students regarding enabling access to technology are crucial to be considered, which is unique and was included in this gerontological nursing competencies framework when developing curricula. The gerontological nursing competencies framework can be integrated into higher education and curriculum development to emphasize levels of gerontological nursing practice [14, 32]. Integrating the gerontological nursing competencies into competency frameworks, learning and development plans, and recruitment of aged-care nurses can ensure the quality of gerontological nursing care [11]. Future research can develop a gerontological nursing competencies workbook and teaching materials to provide evidence-based guidance for improving aged-care education.

There are some limitations of this study that should be acknowledged. First, there could be potential bias when using an online survey method (i.e., selection bias). All items in the survey had to be answered to proceed to the next page. We may have missed some potential respondents who did not complete the survey. However, the strength of using such a technique is that we did not need to deal with missing values. Second, a social-desirability bias is unavoidable; for example, participants may have inaccurately answered about their competencies in certain items, such as legal and ethical aspects. Future evaluations can integrate knowledge and skill tests with a gerontological nursing competencies scale to understand the overall capacity of providing aged care among nursing students. Third, the cross-sectional study design has a limitation that only correlational relationship can be examined. Future studies with a longitudinal design to investigate the casual relationships between variables are warranted. Nevertheless, this study provides insights and recommendations for aged-care education with a representative sample in Taiwan. This study also provides evidence on determinants of the intention to work in aged care.

## Conclusions

Understanding the intention to work in aged care and the level of gerontological nursing competencies among nursing students is vital to support future education, workforce capacity building, and clinical interventions in aged care. The gerontological nursing competency framework and the scale are reliable and valid tools that can be implemented in future research, higher education, and curriculum development. Future research can adopt the gerontological nursing competency framework to improve the quality of aged-care education and facilities. This study also provides insights on determinants of the intention to work in aged care, particularly gerontological nursing competencies.

### Electronic supplementary material

Below is the link to the electronic supplementary material.

Supplementary Material 1

## Data Availability

The data that support the findings of this study are available from the corresponding author upon reasonable request.
