# Supplementary material for: Determinants of the intention to work in aged care: a cross-sectional study to assess gerontological nursing competencies among undergraduate nursing students
Source: BMC Nurs. 2023 Nov 29;22:448. doi: 10.1186/s12912-023-01613-1 (PMC10685655; doi:10.1186/s12912-023-01613-1)
Supplement: Supplementary file 1 — Supplementary Material 1 [file 12912_2023_1613_MOESM1_ESM.docx]

**Supplementary file 1.** Descriptive statistics of gerontology nursing competencies (GNCs) items (*N* = 358)

|  | GNC |
| --- | --- |
| Items and subscales | Mean (SD) |
| 1. **Living well for older people across communities and groups** | 3.63 (0.82) |
| 1. Acknowledgement of the person | 3.70 (0.90) |
| 1. Lifestyle engagement | 3.60 (0.94) |
| 1. Diversity | 3.49 (0.93) |
| 1. Antiageist and antidiscriminatory language and practice | 3.73 (0.94) |
| 1. **Maximizing health outcomes** | 3.60 (0.86) |
| 1. Physiological changes associated with ageing | 3.79 (0.88) |
| 1. Pharmacology in ageing | 3.31 (1.06) |
| 1. Health promotion and health education | 3.78 (0.91) |
| 1. Integrated care | 3.58 (1.03) |
| 1. Assessment and goal planning | 3.56 (1.00) |
| 1. **Communicating effectively** | 3.74 (0.87) |
| 1. Communication as a process and outcome | 3.72 (0.93) |
| 1. Environment | 3.79 (0.92) |
| 1. Culture of enabling communication | 3.71 (0.93) |
| 1. **Facilitating transitions in care** | 3.55 (0.93) |
| Transitions in care | 3.54 (1.00) |
| Health and social networks | 3.55 (0.95) |
| 1. **Facilitating choices within legal and ethical frameworks** | 3.29 (0.98) |
| 1. Legislation | 3.22 (1.08) |
| 1. Advocacy | 3.42 (1.03) |
| 1. Decision making | 3.23 (1.04) |
| 1. **Partnering with family and caregivers** | 3.72 (0.90) |
| 1. Family caregiver needs | 3.73 (0.94) |
| 1. Collaboration | 3.72 (0.94) |
| 1. **Promoting mental health and psychological well-being** | 3.61 (0.92) |
| 1. Grief and loss | 3.66 (0.94) |
| 1. Assessment | 3.63 (1.01) |
| 1. Mental health and psychological well-being interventions | 3.55 (1.00) |
| 1. **Providing evidence-based dementia care** | 3.52 (1.04) |
| 1. Dementia-specific care | 3.57 (1.08) |
| 1. Assessments | 3.53 (1.08) |
| 1. Interventions and evaluations | 3.46 (1.09) |
| 1. **Promoting optimal pain management** | 3.63 (1.03) |
| 1. Assessments | 3.68 (1.04) |
| 1. Interventions and evaluations | 3.58 (1.07) |
| 1. **Providing palliative care** | 3.56 (1.04) |
| 1. Spiritual care | 3.51 (1.12) |
| 1. The palliative approach | 3.60 (1.03) |
| 1. End-of-life care | 3.56 (1.12) |
| 1. **Enabling access to technology** | 3.53 (0.94) |
| 1. eHealth | 3.53 (0.96) |
| 1. Social networks | 3.54 (0.97) |
| 1. Assistive technology | 3.52 (1.02) |
| **Essential** | 3.65 (0.78) |
| **Enhanced** | 3.52 (0.87) |
| **Total score** | 118.11 (26.21) |

***Notes.*** Essential: Core 1, 2, 3, 4, 6; Enhanced: Core 5, 7, 8, 9, 10, 11. SD, standard deviation.
